# Influence of a Single Deuterium Substitution for Protium on the Frequency Generation of Different-Size Bubbles in IFNA17

**DOI:** 10.3390/ijms241512137

**Published:** 2023-07-28

**Authors:** Alexandr Basov, Anna Dorohova, Vadim Malyshko, Arkadii Moiseev, Alexandr Svidlov, Maria Bezhenar, Yury Nechipurenko, Stepan Dzhimak

**Affiliations:** 1 Department of Fundamental and Clinical Biochemistry, Kuban State Medical University, Krasnodar 350063, Russia; son_sunytch@mail.ru (A.B.); intro-3@yandex.ru (V.M.); 2 Department of Radiophysics and Nanotechnology, Kuban State University, Krasnodar 350040, Russia; elkina0131@gmail.com (A.D.); svidlov@mail.ru (A.S.); jimack@mail.ru (S.D.); 3 Laboratory of Problems of Stable Isotope Spreading in Living Systems, Federal Research Center of the Southern Scientific Center of the Russian Academy of Sciences, Rostov-on-Don 344006, Russia; 4 Scientific Department, Kuban State Agrarian University, Krasnodar 350004, Russia; moiseew_a@rambler.ru; 5 Department of Function Theory, Kuban State University, Krasnodar 350040, Russia; mia1610@yandex.ru; 6 Engelhardt Institute of Molecular Biology, Russian Academy of Sciences, Moscow 119991, Russia

**Keywords:** DNA bubbles, open states, deuterium, interferon alpha-17 gene, DNA, rotational movements of nitrogenous bases, dynamics of a double-stranded DNA molecule

## Abstract

The influence of a single ^2^H/^1^H replacement on the frequency generation of different-size bubbles in the human interferon alpha-17 gene (IFNA17) under various energies was studied by a developed algorithm and mathematical modeling without simplifications or averaging. This new approach showed the efficacy of researching DNA bubbles and open states both when all hydrogen bonds in nitrogenous base pairs are protium and after an ^2^H-substitution. After a single deuterium substitution under specific energies, it was demonstrated that the non-coding region of IFNA17 had a more significant regulatory role in bubble generation in the whole gene than the promoter had. It was revealed that a single deuterium substitution for protium has an influence on the frequency generation of DNA bubbles, which also depends on their size and is always higher for the smaller bubbles under the largest number of the studied energies. Wherein, compared to the natural condition under the same critical value of energy, the bigger raises of the bubble frequency occurrence (maximums) were found for 11–30 base pair (bp) bubbles (higher by 319%), 2–4 bp bubbles (higher by 300%), and 31 bp and over ones (higher by 220%); whereas the most significant reductions of the indicators (minimums) were observed for 11–30 bp bubbles (lower by 43%) and bubbles size over 30 bp (lower by 82%). In this study, we also analyzed the impact of several circumstances on the AT/GC ratio in the formation of DNA bubbles, both under natural conditions and after a single hydrogen isotope exchange. Moreover, based on the obtained data, substantial positive and inverse correlations were revealed between the AT/GC ratio and some factors (energy values, size of DNA bubbles). So, this modeling and variant of the modified algorithm, adapted for researching DNA bubbles, can be useful to study the regulation of replication and transcription in the genes under different isotopic substitutions in the nucleobases.

## 1. Introduction

The study of the reproduction mechanisms of genetic information makes it possible to expand the basic knowledge of biological systems and discover useful applications in biomedical research and biotechnology. The essential processes of any living organism are related to the configuration of the DNA macromolecule, and the generation of transcription bubbles during the transfer of genetic information substantially depends on the sequence of its nitrogenous bases [1,2,3,4]. It is well known that in order to copy genetic information, a transcription bubble appears, moving along the DNA strand [5]. A very important influence on transcriptional activity is exerted by the relative lifetimes of DNA denaturation bubbles, which are 12–14 base pairs long for various promoters of different genes [6]. For example, studying the distribution of bubble lifetimes and their length in DNA at physiological temperature, carried out in this work [7], showed that there is a relationship between the melting temperature and the content of Guanine–Cytosine (G–C) in the DNA sequence and also found that the average bubble lifetime decreases with the increase in either bubble length or G–C content. In this case, short bubbles have similar probabilities for any content of G–C, but longer bubbles are significantly more likely to occur in sequences that are rich in Adenine–Thymine (A–T).

A particularly important issue is to investigate the role of different-sized DNA bubbles in biological processes since not only the local DNA sequence but also the local shape of DNA affects both the intensity of its interaction with proteins and the molecular deformation of DNA due to DNA–protein binding [8]. Considering the fact that the experimental study of the spatial structure of DNA still faces a number of problems, the most important of which is the limitation of the dimensional resolution of the available biophysical tools [9], the partial destabilization of double-stranded DNA experiencing spontaneous local conformational fluctuations (or “fraying”), it is possible to study at the present time by various other methods, the key of which is mathematical or physical modeling [10].

For the first time, such an approach was provided in [11], and subsequent works made it possible to develop a mechanical model for describing the dynamics of DNA, taking into account various forms of external influence, many effects of dissipation, and the heterogeneity of the molecule [12]. For instance, in one of the articles [13], the effect of DNA torque on the movement of transcription bubbles in the potential field formed by the PTTQ18 plasmid sequence was numerically studied. It was found that the stationary rates of the bubbles significantly depend on the value of the torque moment and do not depend on the initial velocities of the bubbles. Thus, by changing the moment of torsion, it is possible to make the transcription bubbles move at a given stationary rate [13]. Another well-known model for describing DNA melting is the Payrard–Bishop–Doxois (PBD) model [14,15], which, however, does not take stacking into account and, when used, there is a discrepancy by several orders of magnitude in the equilibrium constant in the local unwinding reaction from experimental data [16]. It is also possible to study DNA bubble dynamics in terms of a Fokker–Planck equation on the basis of the free energy in the Poland–Scheraga model [17,18].

Additionally, it is noted that the functional activity of a molecule depends on its properties, such as elasticity and length, and these parameters can change under the influence of external regulatory forces. Up-to-the-minute techniques allow these forces to be manipulated at the microscopic level [19,20], giving insight into the hydrogen bonds that hold the strands together. For example, the elastic properties of DNA, flexibility, and stretch along the direction of the external force are associated with fluctuations in the number of bubbles and the average number of bubbles, respectively [21]. As the filaments approach each other, the tensile force primarily contributes to the formation of bubbles, while the average bubble length increases with the tensile force, reaching a peak near the transition point and then fading away as it gradually collapses into a single filament. It is shown that the bubbles under the action of a tensile force, on average, increase in comparison with the melting process [22]. 

Also, based on the angular model, it was found that the presence of deuterium in the chain of nucleotides can cause, depending on the value of the hydrogen bond-breaking energy, both an increase and a decrease in the probability of the occurrence of open states [23]. Moreover, the isotopic composition of the media, causing primarily a single ^2^H/^1^H replacement, affects the size of denaturation bubbles and the probability of their formation [24]. The angular model of DNA [25,26] makes it possible to take into consideration a quite wide range of external influences, such as the viscosity of the medium, the isotopic composition, and external forces [27,28,29,30]. In addition, stacking is also contemplated in the coefficient responsible for the elastic properties of the thread in this model. All of this is extremely important since stacking has a major effect on the stability of the base pair and is the predominant force holding the double helix together, and stacking strongly affects both the functional efficiency of the molecule and the accuracy of DNA synthesis [31,32]. 

In our study, we modeled the influence of single ^2^H/^1^H replacement on the frequency generation of different-size bubbles in the gene encoding human interferon alpha 17, which enables cytokine activity and type I interferon receptor binding activity. It matters a lot in several essential processes, including changes in the morphology and behavior of the cells involved in immune response, activation of B lymphocytes, positive regulation of peptidyl-serine phosphorylation of signal transducer and activator of transcription protein, the role (for some gene variants) in chronic immune thrombocytopenia pathogenesis, and the changing risk of cervical squamous cell carcinoma and prostate cancer [33,34,35,36]. Therefore, researching the IFNA17 expression mechanisms, including the impact of the isotopic exchange on the DNA bubble generation, can provide useful data for the development of algorithms for gene suppression or activation in patients with predicted severe diseases. 

The aim of this work is to study the effect of a single deuterium substitution on the frequency generation of different-size bubbles in IFNA17 under varied energies that is based on the mechanical model of DNA without simplifications or averaging while also considering the contribution of the AT/GC ratio. 

## 2. Results

**Figure 1:**
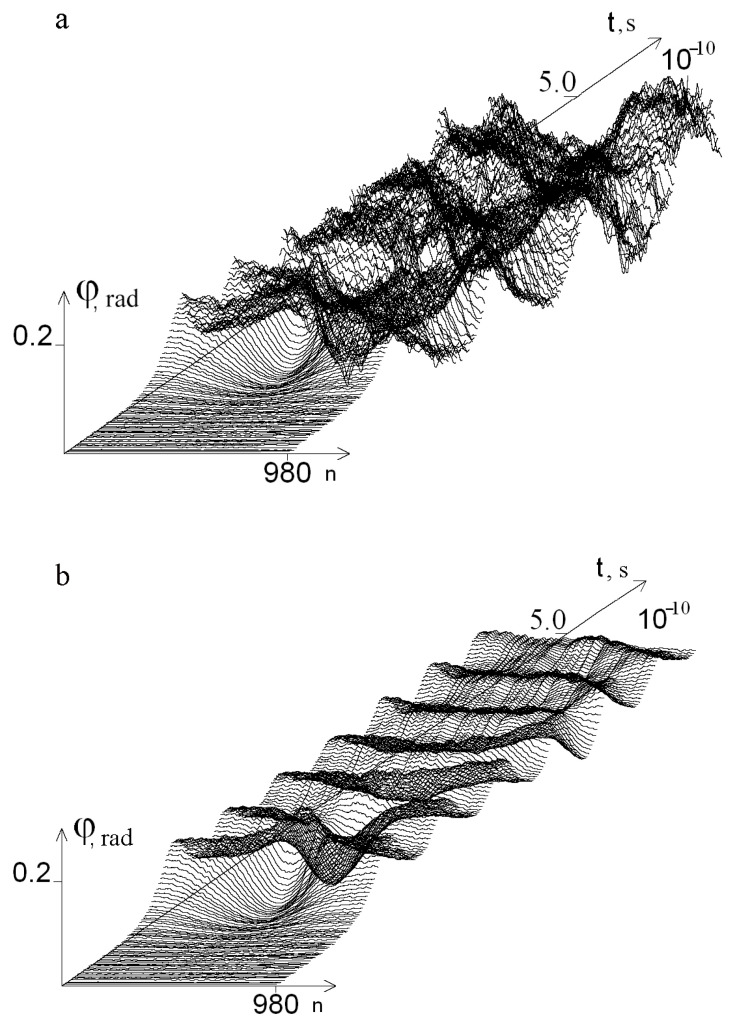
Graphs of angular deviations of the 1-st chain nitrogenous bases of IFNA17 over a period of time: [0, t = 5.0 × 10^−10^ s], under $E_{cr}$ equals 0.250·10^−22^ N·m (**a**) and $E_{cr}$ equals 0.600·10^−22^ N·m (**b**).

The graphs of the angular deviations of the 1-st chain nitrogenous bases in the gene encoding interferon alpha 17 under the first $E_{cr}$, which equals 0.250·10^−22^ N·m, over a period of time from 0 to 5.0 × 10^−10^ s, are presented in Figure 1a. In comparison to this data, the graphs of the angular deviations show the same chain of DNA molecules, but when every $P_{i}$ equals 0.0 for all bubble groups and open states (OS) under 0.600·10^−22^ N·m over a similar period of time, as shown below in Figure 1b.

**Figure 2:**
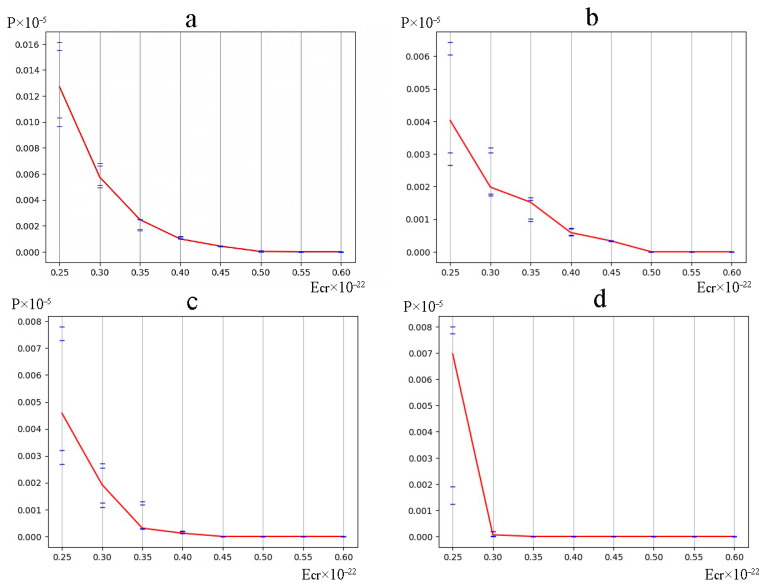
Dynamics of the different-size DNA bubble occurrence ((**a**) 2–4 bps, (**b**) 5–10 bps, (**c**) 11–30 bps, (**d**) over 30 bps) in the gene encoding interferon alpha 17 under natural conditions and after a single ^2^H/^1^H replacement (with gradation of the bubble occurrence frequency by modified BJ algorithm). Note: for each energy, the 1st (highest) cross dash is $P_{i_{max}}$, the 2nd cross dash is the bottom of the range “Maximum”, the 3rd cross dash is the top of the range “Minimum”, and the 4th cross dash is $P_{i_{min}}$; the red line is the DNA bubble occurrence frequency under the condition that all hydrogen bonds in IFNA17 are ^1^H ($P_{0}$), bps are nitrogenous base pairs.

**Figure 3:**
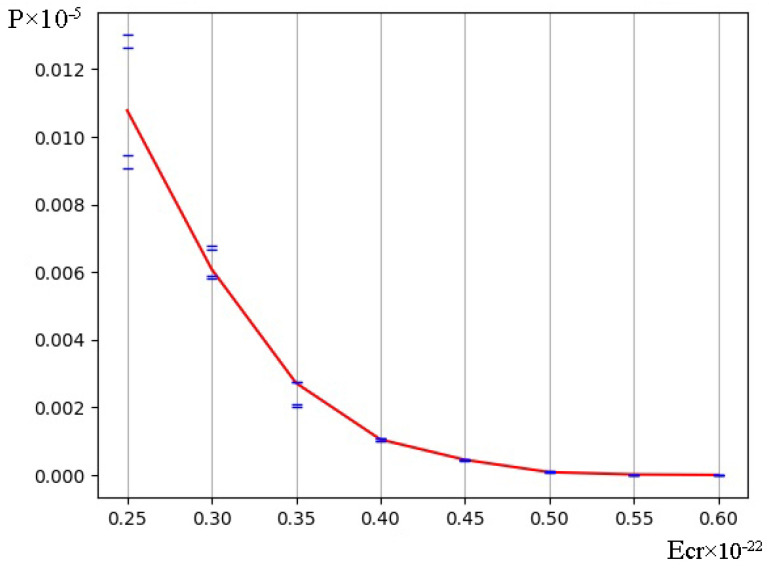
Dynamics of the open state (OS) occurrence in the gene encoding interferon alpha 17 under natural conditions and after a single ^2^H/^1^H replacement (with gradation of the OS occurrence frequency by the modified BJ algorithm). Note, for each energy: the 1st (highest) cross dash is $P_{i_{max}}$, the 2nd cross dash is the bottom of the range “Maximum”, the 3rd cross dash is the top of the range “Minimum”, and the 4th cross dash is $P_{i_{min}}$; the red line is the OS occurrence frequency under the condition that all hydrogen bonds in IFNA17 are ^1^H ($P_{0}$).

The occurrence frequency for each DNA bubble group was counted by using the above-mentioned variant of the modified BJ algorithm, according to which in Figure 2, the dynamics of their occurrences in the studied gene are rendered under both natural conditions and after a single ^2^H/^1^H substitution. From the obtained data, it is obvious that the bubble occurrence frequency in the human interferon alpha-17 gene (IFNA17) under conditions where all hydrogen bonds in nucleotides are ^1^H has an inverse relationship with the bubble size in these groups. This tendency is also confirmed by the results of the evaluation of the OS occurrence frequency (Figure 3); $P_{0}$ is always higher than the $P_{0}$ of each bubble group under the same energy.

It should be noted that the natural frequency of small DNA bubble occurrence is permanently the highest compared with *P*_0_ of 2, 3, and 4 bubble groups (at least by 29.5% more than *P*_0_ of metastable bubbles under $E_{cr}$ equals 0.450·10^−22^ N·m, and far more compared with others), but it has a little difference (by 17.7% or less) from *P*_0_ of OS throughout the energy range of 0.250·10^−22^ N·m to 0.450·10^−22^ N·m (henceforth from 0.250 to 0.450). Moreover, the larger the bubble size, the lower the $E_{cr}$, under which its *P*_0_ equals 0.0: for small bubbles–0.550; for metastable bubbles–0.500; for large bubbles–0.450; and for very large bubbles–0.350 (Figure 2). For comparison with the previous values, higher energy (above 0.550) is required to achieve the zero value of natural OS frequency occurrence (Figure 3). After a single ^2^H/^1^H substitution, the changes to $P_{0\;}$ are almost as similar to $P_{i_{max}}$ for all DNA bubble groups and OS that were observed, and the smallest differences between $P_{0\;}$ and $P_{i_{max}}\;$ under the same conditions occurred in the 0.350–0.450 energy diapason.

Albeit the fluctuations of absolute $P_{i_{min}}\;$ values were less significant compared with $P_{i_{max}}$ changes, but the relative $P_{i_{min}}\;$ differences between bubble groups were strong and higher by 55.6–573.9% in the energy range 0.250–0.400 (Figure 2). In all cases, it was found that the small DNA bubbles had the highest $P_{i_{min}}$ values in comparison with other bubble groups (at least by 27.0% under $E_{cr}\;$ equal to 0.450), and additionally, these did not differ much from the similar OS frequency occurrence (less than 18.2% throughout the 0.250–0.450 energy interval).

**Figure 4:**
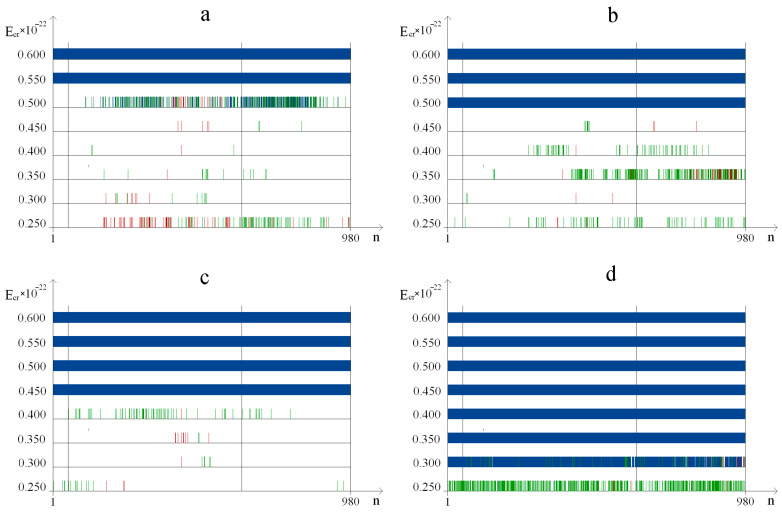
Distribution of nucleobase pairs in the three parts of IFNA17 leading, after a single ^2^H/^1^H replacement, to extreme frequencies of different-size DNA bubble occurrences ((**a**) 2–4 bps, (**b**) 5–10 bps, (**c**) 11–30 bps, (**d**) over 30 bps). Note: the red dot is the location of the deuterium atom in the gene, which leads to the maximum probability of DNA bubble occurrence (“Maximum” range); the green dot is the location of the deuterium atom in the gene, which leads to the minimum probability of DNA bubble occurrence (“Minimum” range, $P_{i}$ > 0.0); and the dark-blue dot is the location of the deuterium atom in the gene, which leads to the probability of 0.0 for a DNA bubble occurrence (close states, $P_{i}$ = 0.0).

**Figure 5:**
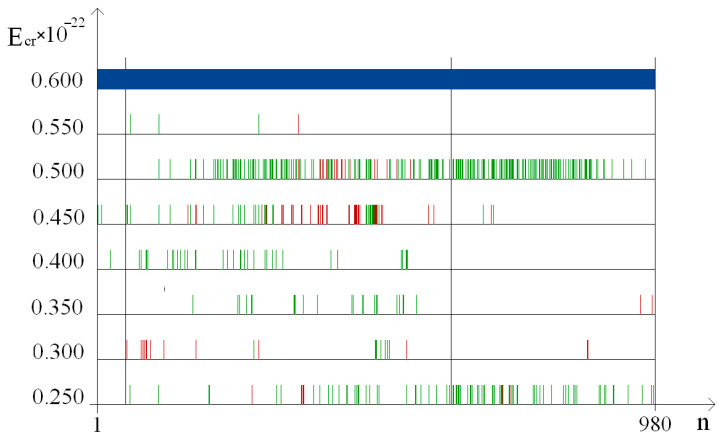
Distribution of nucleobase pairs in the three parts of IFNA17, leading after a single ^2^H/^1^H replacement, to the extreme frequencies of OS occurrences (1 bps). Note: the red dot is the location of the deuterium atom in the gene, which leads to the maximum probability of OS occurrence (“Maximum” range); the green dot is the location of the deuterium atom in the gene, which leads to the minimum probability of OS occurrence (“Minimum” range, $P_{i}$ > 0.0); and the dark-blue dot is the location of the deuterium atom in the gene, which leads to the probability of 0.0 for an OS occurrence (close states, $P_{i}$ = 0.0).

In Figure 4, the three parts of IFNA17 showed a distribution of nucleobase pairs leading to the maximum and minimum probability of different DNA bubbles and OS occurrences after a single ^2^H/^1^H replacement under a 0.250–0.600 energy diapason. It is obvious from the graphs that the promoter region has no nucleotides in the range “Maximum” under the overwhelming majority of studied $E_{cr}$ (from 0.300 to 0.500) values for any DNA bubble group or OS (Figure 4 and Figure 5).

In comparison to the promoter and termination sequences, the coding region has the highest sum number of the nucleotides from the “Maximum” range (p$\chi_{Yates}^{2}$ = 0.0873, p$\chi_{Yates}^{2}$ < 0.0001, respectively), which reinforces OS and bubble generation in different groups foremost under $E_{cr}$ equals 0.250 (Figure 4, Table 1). At the same time, in the coding region, the maximum value of the nitrogenous bases was obtained, including in the “Minimum” range (p$\chi_{Yates}^{2}$ = 0.0002 and p$\chi_{Yates}^{2}$ < 0.0003 compared with the promoter and termination sequence, respectively; Figure 4, Table 2). In addition, more nucleobases from these ranges were in the III part as opposed to the I part of the gene: p$\chi_{Yates}^{2}$ = 0.0014 (for “Maximum”) and p$\chi_{Yates}^{2}$ < 0.0001 (for “Minimum”). It should be noted that the differences between AT fractions of the two ranges “Maximum” and “Minimum” in the coding region (39.9% and 44.5%) and the termination sequence (75.0% and 67.5%) were significant (p$\chi_{Yates}^{2}$ = 0.0004 and p$\chi_{Yates}^{2}$ < 0.0001). 

**Figure 6:**
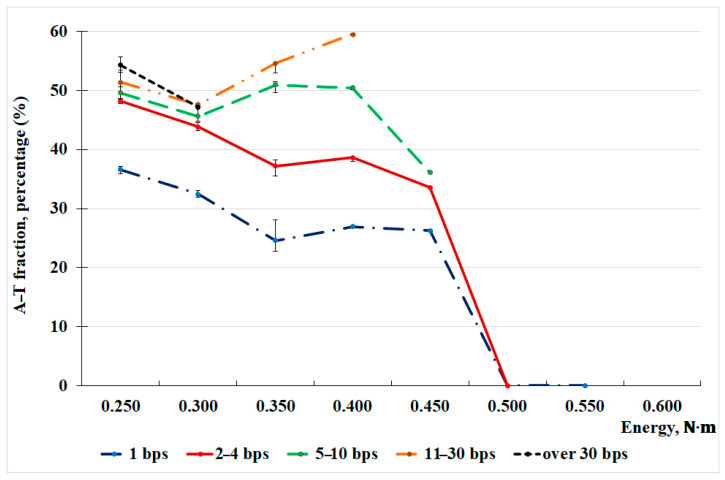
A–T/G–C ratio of the OS and different DNA bubbles after a single ^2^H/^1^H replacement throughout 0.250–0.600 energy diapason in IFNA17. Note: Energy = x × $10^{-22},\;N\cdot m$ (where x can be 0.250, 0.300, 0.350, 0.400, 0.450, 0.500, 0.550, or 0.600), each dot is the median, each upper cross dash is the 75th percentile, and each lower cross dash is the 25th percentile.

Moreover, the promoter of IFNA17 has the lowest sum of $n_{max}$ and $n_{min}$ compared to the similar values of the coding region and termination sequence: p$\chi_{Yates}^{2}$ < 0.0002 and p$\chi_{Yates}^{2}$= 0.0013, respectively. It is worth noting that the least A–T/G–C ratio fluctuation among the five $n_{max}$ sums of the OS and each bubble group in the whole energy diapason was in the coding region (minimum 28.6% A–T for $n_{max}$ of the group 3, maximum 46.2% A–T for $n_{max}$ of the group 4, Table 1). And the least A–T/G–C ratio fluctuation among the five $n_{min}$ sums in the same studied groups was in the termination sequence of IFNA17 (minimum 41.7% A–T for $n_{min}$ of the group 3, maximum 70.8% A–T for $n_{min}$ of the group 4, Table 2). Studying the nucleobases forming DNA bubbles and OS in the gene after a single ^2^H/^1^H replacement revealed the obvious role of the A–T/G–C ratio in the three following dependencies (Figure 6):

- The larger the number of dissociated adjacent hydrogen bonds, the higher the percentage of A–T base pairs among them in the 0.250–0.450 energy interval: Spearman’s rank correlation coefficients are positive and increase from 0.877 (*p* < 0.001, $E_{cr}$ = 0.300) to 0.968 (*p* < 0.001, $E_{cr}$ = 0.400);
- The higher the $E_{cr}$ (in the 0.250–0.450 energy range), the lower the percentage of A–T base pairs in the OS, and bubble groups that have the same sizes (with the exception of the large bubbles: $r_{\mathrm{Spearman}}=$ 0.650, *p* < 0.001): Spearman’s rank correlation coefficients are negative and change from −0.321 (*p* < 0.001, metastable bubbles) to −0.902 (*p* < 0.001, small bubbles);
- If the $E_{cr}$ equals 0.500 or more, the percentage of A–T base pairs in OS or small bubbles is equal to 0.0.

**Figure 7:**
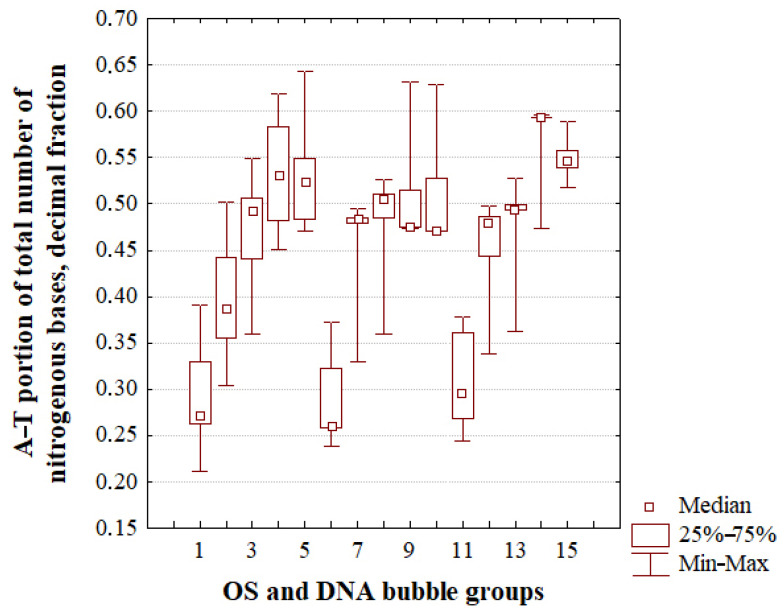
A–T fractions of the different DNA bubbles and OS after a single 2H/1H isotopic substitution throughout the 0.250–0.450 energy range in IFNA17. Note: 1, 6 and 11 are OS; 2, 7 and 12 are bubble group 1; 3, 8 and 13 are bubble group 2; 4, 9 and 14 are bubble group 3; 5, 10 and 15 are bubble group 4; 6–10 were generated by nucleobases from the “Maximum” range; 11–15 were generated by nucleobases from the “Minimum” range; and 1–5 were generated by nucleobases located out of both the “Maximum” and “Minimum” ranges.

Also, in the 0.250–0.450 energy range, the higher dispersion values of the A–T/G–C ratio in the OS and three bubble groups were observed after a single ^2^H/^1^H substitution under $E_{cr}$ equals 0.350 (Figure 6). In the majority of cases, no significant changes were found in the data on the content of AT in the bubble groups of the same size and OS generated by the sum nucleobases from the “Maximum” and “Minimum” ranges throughout the 0.250–0.450 energy diapason (Figure 7). However, small bubbles generated by these nitrogenous bases have significantly more A–T (by 24.3% and 24.8%, respectively) than the bubbles of the same size, which were formed by the base pairs located out of the “Maximum” or “Minimum” ranges ($p\chi_{B}^{2}$ < 0.0001, $p_{KWt}$ < 0.0001). The metastable bubbles with the slightly higher A–T percentage (by ~ 2.6% compared to the data of nucleobases located out of both ranges) were produced by the sum of $n_{max}$ ($p\chi_{B}^{2}$ < 0.0001, $p_{KWt}$ = 0.0091).

For gene regulation, the dependencies of the number of changes of the dissociated H-bonds at specific energies when the $P_{i}$ of parts of the nucleobases that generate certain DNA bubbles equals 0.0 (close state) can have a large significance:

- At first, the higher the $E_{cr}$ (in the 0.250–0.550 energy range), the lower the number of dissociated adjacent hydrogen bonds, so that the energy values, when the $P_{i}$ equals 0.0 for all base pairs in each part of IFNA17, have an inverse relationship with bubble sizes (Table 2, Figure 4);
- Second, concerning very large bubbles in the termination sequence, ^2^H-substituted G–C bases (77.1% of all G–C) are closed under 0.300 energy more easily than ^2^H-substituted A–T bases (59.4% of all A–T, p$\chi_{Yates}^{2}$ < 0.0001, Figure 4);
- In the third place, different from the promoter, which has no close states for the 1 bubble group under 0.500 energy, in the coding region and termination sequence (in total 931 bps), ^2^H-substituted G–C bases (30.8% of all G–C) are closed easier than ^2^H-substituted A–T bases (16.8% of all A–T, p$\chi_{Yates}^{2}$ < 0.0002, Figure 4).

All in all, the A–T richest III part of IFNA17 has the highest percentage of ^2^H-substituted G–C bases, which leads most significantly to a decrease in the occurrences of groups 1 and 4 bubbles (under $E_{cr}$ equal to 0.500 and 0.300, respectively) because of the rise of the corresponding non-dissociated adjacent hydrogen bonds in the whole gene (Supplementary Materials).

## 3. Discussion

The participation of deuterium atoms in the formation of hydrogen bonds in a DNA molecule can provide a significant change in the time of genetic information transmission, which suggests that even a slight isotopic shift in the environment can affect the rate of metabolic processes [37,38,39,40,41,42,43,44,45,46,47,48,49,50]. The carried out studies on the ^2^H/^1^H isotope exchange effect in hydrogen bonds between pairs of nitrogenous bases on the inequality distribution of the probabilities of open states along the length of the gene showed that an external action in the frequency range from 10^11^ s^−1^ to 10^8^ s^−1^ causes vibration of the DNA molecule with a frequency different from the frequency of external influence.

It is obvious that one of the obstacles to the study of DNA is the repeated modeling of its molecular dynamics using various simplifying assumptions, including the averaging of characteristics in heterogeneous chains of nucleobases and the simplification of the nature of the bond between complementary nitrogenous bases, which sharply reduces the reliability of the resulting data when such a system of equations is used. So, to describe the open states, the sine-Gordon equation and its particular solution, called “kink”, are frequently used [51,52]. Such an approach has a number of shortcomings. At first, the bond breaking corresponds to angular deviations of 180° from the equilibrium position, so it is not clear how the “closing” of breaks can be described using “kinks”. Secondly, the sine-Gordon equation models a simplified coupling in base pairs of pendulums, which leads to a significant change in the nature of the solution with a decrease in the amplitude of angular vibrations of nucleobases by about 100 times [53]. In third place, there is another drawback to this model [12], which is that fluctuations of bases in extreme pairs are not taken into account. In its turn, another well-known Peyard–Bishop–Doxois (PBD [14,15]) model describes DNA melting quite well, but when it is used to describe the local denaturation of the nitrogenous base pairs under subcritical temperatures, a number of problems arise, which are different from experimental data by several orders of magnitude in the mismatch of the equilibrium constant in DNA local unwinding reactions [16]. 

In contradistinction to the models presented above, the soliton mechanism of bioenergy transfer in DNA shows [54,55] that the emerging nonlinear interaction that accompanies the distribution of vibrational quanta along the macromolecule is via vibrational excitations (excitons) and acoustic phonons, which leads to the formation of a soliton spreading along the chain of the nucleic acid.

However, this model has a disadvantage, which is that the solution lifetime is very short (from 10^−13^ to 10^−12^ s) under temperatures close to natural conditions, resulting in the inapplicability of this approach to the study of DNA molecular dynamics in biological systems [56].

In the present work, a modified mathematical model is considered that does not have the above-described disadvantages, using the similarity between a DNA molecule and a mechanical system consisting of two chains of interconnected pendulums. Therefore, the dynamics of the DNA molecule were studied due to the rotational movement of nitrogenous bases around the pentose-phosphate two-stranded backbone without simplifications or averaging. Moreover, a new variant of the algorithm for counting the number of different-sized bubbles was applied, which is advisable to use to research the functional activity of DNA. The latter is due to the vital role of DNA bubbles of different sizes in the implementation of the various specific nucleic acid functions [57,58,59]. For instance, a thermally driven process called DNA “breathing” that stems from the regeneration of the metastable bubbles [60,61], leads to the transient adoption of local conformations of nucleic acid significantly different from its most stable structures. And the fluctuations of the sugar–phosphate backbones that arise due to the mechanistically useful local base pair opening reactions allow some proteins of genome expression (first of all polymerases) to require access to single-stranded DNA more effectively [62,63]. It well known that the AT-richest DNA sequences (usually of 30–100 base pairs [64,65,66]) have the preferential ability to unwind the double helix, which is essential for DNA replication and transcription under natural conditions [67]. Nevertheless, under a specific $E_{cr}$ after a single ^2^H-substitution in the termination sequence, which is the richest in AT base-pairs compared to the I and II parts of IFNA17, it has the highest percentage of ^2^H-substituted G–C bases, which significantly decreases the occurrence of bubbles in groups 1 and 4. And the last phenomenon can significantly slow down the unwinding of DNA and its interaction with proteins, despite the highest percentage of A–T nucleobases in this part of the gene. Thus, our modeling of the occurrence of different-size DNA bubbles after an ^2^H-substitution in certain parts of IFNA17 obviously demonstrates the possibility of a significant change in the functional activity of the gene under specific values of energy. Moreover, the described changes apply not only to groups of small and metastable bubbles but also to very large bubbles of group 4, which can have a special effect on the dynamic pattern of DNA–protein interactions, especially with a longer lifetime [3,68,69,70]. Wherein, the slowdown in DNA bubble relaxation can be realized via several possible mechanisms, including the generation of hairpin loops, base stacking in a single-stranded domain, mismatched double-stranded domain reclosure, mechanically constrained situations forming the torque, and others [3,71,72], which can also be, apparently, affected by single isotopic substitutions.

In addition, it was shown earlier that, differently from the longer bubbles, the small ones with any GC content have roughly the same likelihood of occurrence under natural conditions, whereas the big bubbles predominantly occur in AT-rich sequences of DNA [7]. Albeit, after a single ^2^H/^1^H replacement, our study approved the above-mentioned statement for all bubble groups under less energy values (from 0.250 to 0.450, Figure 6), the group 1 bubbles, generated via nucleobases pertaining to the “Maximum” or “Minimum” ranges, have a higher A–T ratio (by 24.3% and 24.8%, respectively) than the bubbles of the same size, which were formed by the base pairs located out of the “Maximum” or “Minimum” diapasons.

Additionally, under $E_{cr}$ values equal to 0.500, there was an abrupt decrease in the A–T ratio (down to 0.0) in small bubbles and OS. Furthermore, our work demonstrated an inversely significant relationship between the A–T ratio in the different bubbles and $E_{cr}$ (with the exception only of the bubbles of group 3 throughout the energy diapason from 0.300 to 0.400). The ability of the bubbles of size 11 to 30 bps to remain stable with the higher content of AT under higher energy values can be explained by short-range attractive interactions within nucleobases in combination with electrostatic interactions at the Debye–Hückel level [59]. During the replication process, the described effect can also be enhanced because the DNA-binding proteins interact with unpaired bases within a bubble to assist in the stability of large and very large bubbles [72]. Also, a single ^2^H-substitution can alter the structure and activity of nucleic acids by changing base-pairing interaction strength due to the difference not only in the geometry, stability, and dissociation energy of ^1^H- and ^2^H-bonds but also via the inequality of their vibrational zero-point energies [39,40,50,73,74,75,76,77,78,79]. The decreasing energy barrier caused by the zero-point vibration of the DNA hydrogen bond will promote interaction between nucleic acid and protein, both generating new bubbles and stabilizing the large and very large ones. At the same time, an ^2^H/^1^H-substitution initiates the reduction of the oscillations (or it creates conditions for a lower vibrational frequency) due to the higher mass isotope effect [80,81], which can slow down the bubble formation in the gene, thereby disrupting transcription and replication processes. Especially, forming DNA CSNBs is absolutely inappropriate for the implementation of the essential processes in cells because of the creation in the gene of many excessively unbreakable sections, which completely prevent any interaction of DNA with other biomolecules. Therefore, under a specific energy range, even slight isotope fluctuations can lead to significant changes in the molecular dynamics of nucleic acids. Moreover, various water isotopologues can additionally affect the dynamics of bubble conformation and their lifetimes, wherein the water associated with DNA directly not only has a larger local viscosity than in solution but also stronger hydrogen bonds under heavier isotopic composition [42,68]. Although, under natural conditions, the DNA zones of higher stability to denaturation are commonly relevant to non-coding regions [82,83], our study showed that even a single ^2^H/^1^H replacement in the III part of IFNA17 creates more $n_{\max}$ and $n_{\min}$ than their sum after an ^2^H-substitution in the promoter (p$\chi_{Yates}^{2}$ = 0.0013, Table 1, Table 2), which indicates an important role of non-coding gene regions in the regulation of the DNA bubble occurrences in its different parts. Based on this, it can be assumed that regulation of DNA denaturation is a far more complex process than only through classical transcription initiation points (e.g., TC- and TATA-motifs and others [84]). In addition, due to the developed model and new variant of the modified BJ algorithm, after a single ^2^H-substitution, it was possible to reveal both the impact of $E_{cr}$ (negative in the diapason from 0.250 to 0.450) and the bubble sizes (positive for the ones 10 bps and less) on the AT/GC ratio in DNA bubbles. These data point out not only the significance of the AT/GC ratio for the initiation of the DNA denaturation process but also the importance of this ratio for bubble lifetimes and the stability of their particular groups. Furthermore, using such a mathematical approach, it is possible to reduce the proportion of both false positive and false negative results in the analysis of the intensity of hydrogen bond dissociation when studying DNA molecular dynamics [85,86].

In general, on the basis of this study, below are presented the clues and inferences of this work, providing in detail the confirmation for further conclusions:

- The positive values of bubble occurrence frequencies in IFNA17, in all cases under the condition that all hydrogen bonds in nitrogenous base pairs are ^1^H, have an inverse relationship with the DNA bubble size throughout the energy range of $0.250\times 10^{-22}$ N·m to $0.500\times 10^{-22}$ N·m: $P_{0\;of\;group\;1}$ > $P_{0\;of\;group\;2}$ > $P_{0\;of\;group\;3}$ >$\;P_{0\;of\;group\;4}$ (Figure 2).
- The frequency of bubble occurrences in the gene under natural conditions decreases progressively with rising $E_{cr}$, reaching zero with an inverse relationship to DNA bubble size: the larger the size of the bubbles, the lower the energy under which their *P*_0_ equals 0.0, and when $E_{cr}$ equals $0.500\times 10^{-22}$ N·m, the $P_{0}$ equals 0.0 for all bubble groups (Figure 2).
- When H-bonds only have protium in IFNA17, the occurrence frequency of small bubbles is even higher than the value of OS under $0.250\times 10^{-22}$ N·m energy and, in the diapason from $0.300\times 10^{-22}$ N·m to $0.450\times 10^{-22}$ N·m, the data did not differ significantly compared to each other (less than 9.2% (Figure 2a and Figure 3).
- After a single ^2^H/^1^H replacement under the 0.250–0.450 energy interval, small DNA bubbles have the highest $P_{i_{max}}$ and $P_{i_{min}}$ when compared to other bubble groups, and these values did not differ much in similar OS frequency occurrences (less than 24.0% and 18.2% at their peaks, respectively, Figure 2 and Figure 3).
- Different from the promoter and termination sequences, the coding region has the highest total $n_{max}$ and $n_{min}$ after a single isotopic ^2^H/^1^H substitution (Table 1 and Table 2), which indicates its strong regulatory significance in both the acceleration and retardation of DNA bubbles and OS generation in the whole gene.
- In the II part of IFNA17, both ranges “Maximum” and “Minimum” contained predominantly G–C base pairs (60.1% and 55.6%, respectively), which significantly differed from similar G–C fractions (25.0% and 32.5%, respectively) in the III part of the gene (Table 1 and Table 2).
- Under $E_{cr}$ values equal to 0.500 or more, after a single ^2^H/^1^H replacement, the formation of small bubbles and OS occurs only via guanine and cytosine nitrogenous bases (Figure 6).
- After a single isotopic ^2^H/^1^H substitution in the 0.250–0.450 energy interval, the A–T/G–C ratio of DNA bubbles and OS has a strong direct correlation with the number of dissociated adjacent H-bonds and an inverse and significant relationship with $E_{cr}$ (the latter has the exception of only bubbles of size 11 to 30 bps, which have a Spearman’s rank correlation coefficient of 0.650, *p* < 0.001, Figure 6).
- The promoter of IFNA17 has the lowest sum of $n_{max}$ and $n_{min}$ in comparison to II (p$\chi_{Yates}^{2}$ < 0.0002) and III (p$\chi_{Yates}^{2}$ = 0.0013) parts of the gene (Table 1 and Table 2); therefore, it has the least impact on both the acceleration and slowdown of the H-bond dissociation after a single ^2^H/^1^H replacement in the whole studied energy range.
- For small and very large bubbles, after a single ^2^H/^1^H replacement at the nucleotide parts of IFNA17 (21.3% and 81.5%, respectively), the occurrence frequencies reached zero values under lower energy in comparison to the $E_{cr}$ for the same bubble groups under natural conditions, so their $P_{0}$ remained above 0.0, but their $P_{i}$ (named $P_{i_{min}}$) were equal to 0.0 under $E_{cr}\;$ values of 0.500 and 0.300, respectively (Figure 2, Supplementary Materials).
- Although the III part of IFNA17 is most depleted in G–C nitrogenous bases (only 29.09% of the total base number), it has the highest percentage of ^2^H-substituted G–C bases, which abruptly decreases the number of small and very large bubbles under $E_{cr}$ equals 0.500 and 0.300, respectively (Supplementary Materials).

Therefore, the above-presented results showed the efficacy of the mathematical modeling and BJ algorithm; nevertheless, our study had some limitations. Foremostly, the data had been obtained with the frameworks of the modeling based on Newton’s equations and representative of the Cauchy problem for the system of 2n ordinary differential equations, without contemplating the whole problem of the “DNA–protein” interaction [1,18,87,88,89], which can dramatically change the bubble lifetimes but differently for each group. However, we do not consider these limitations an insurmountable obstacle and regard the variant of the modified BJ algorithm, adapted for DNA bubbles, as an appropriate method that can be colligated and used with other different complex models investigating the molecular dynamics of nucleic acids.

## 4. Materials and Methods

### 4.1. Mathematical Model

To describe the mechanical movements of nitrogen bases, we used the model that was described earlier [53]. It includes the equations of angular vibrations:

(1) $$I_{1}^{i}\frac{d^{2}\varphi_{1}^{i}\left(t\right)}{dt^{2}}=K_{1}^{i}\left[\varphi_{1}^{i-1}\left(t\right)-2\varphi_{1}^{i}\left(t\right)+\varphi_{1}^{i+1}\left(t\right)\right]\;-\delta^{i}\left(k_{12}^{i}R_{1}^{i}\left(R_{1}^{i}+R_{2}^{i}\right)\sin\varphi_{1}^{i}+k_{12}^{i}R_{1}^{i}R_{2}^{i}\sin\left(\varphi_{1}^{i}-\varphi_{2}^{i}\right)\right)+\;F_{1}^{i}\left(t\right),\;i=\bar{2,n-1},$$

(2) $$I_{1}^{1}\frac{d^{2}\varphi_{1}^{1}\left(t\right)}{dt^{2}}=K_{1}^{1}\left[\varphi_{1}^{2}\left(t\right)-\varphi_{1}^{1}\left(t\right)\right]\;-\delta^{i}\left(k_{12}^{1}R_{1}^{1}\left(R_{1}^{1}+R_{2}^{1}\right)\sin\varphi_{1}^{1}+k_{12}^{1}R_{1}^{1}R_{2}^{1}\sin\left(\varphi_{1}^{1}-\varphi_{2}^{1}\right)\right)+F_{1}^{1}\left(t\right),$$

(3) $$I_{1}^{n}\frac{d^{2}\varphi_{1}^{n}\left(t\right)}{dt^{2}}=K_{1}^{n}\left[\varphi_{1}^{n-1}\left(t\right)-\varphi_{1}^{n}\left(t\right)\right]\;-\delta^{i}\left(k_{12}^{n}R_{1}^{n}\left(R_{1}^{n}+R_{2}^{n}\right)\sin\varphi_{1}^{n}+k_{12}^{n}R_{1}^{n}R_{2}^{n}\sin\left(\varphi_{1}^{n}-\varphi_{2}^{n}\right)\right)+F_{1}^{n}\left(t\right),$$

(4) $$I_{2}^{i}\frac{d^{2}\varphi_{2}^{i}\left(t\right)}{dt^{2}}=K_{2}^{i}\left[\varphi_{2}^{i-1}\left(t\right)-2\varphi_{2}^{i}\left(t\right)+\varphi_{2}^{i+1}\left(t\right)\right]\;+\delta^{i}\left(k_{12}^{i}R_{2}^{i}\left(R_{1}^{i}+R_{2}^{i}\right)\sin\varphi_{2}^{i}-k_{12}^{i}R_{1}^{i}R_{2}^{i}\sin\left(\varphi_{2}^{i}-\varphi_{1}^{i}\right)\right)\;+F_{2}^{i}\left(t\right),\;i=\bar{2,n-1},$$

(5) $$I_{2}^{1}\frac{d^{2}\varphi_{2}^{1}\left(t\right)}{dt^{2}}=K_{2}^{1}\left[\varphi_{2}^{2}\left(t\right)-\varphi_{2}^{1}\left(t\right)\right]\;+\delta^{i}\left(k_{12}^{1}R_{2}^{1}\left(R_{1}^{1}+R_{2}^{1}\right)\sin\varphi_{2}^{1}1-k_{12}^{1}R_{1}^{1}R_{2}^{1}\sin\left(\varphi_{2}^{1}-\varphi_{1}^{1}\right)\right)+F_{2}^{1}\left(t\right),$$

(6) $$I_{2}^{n}\frac{d^{2}\varphi_{2}^{n}\left(t\right)}{dt^{2}}=K_{2}^{n}\left[\varphi_{2}^{n-1}\left(t\right)-\varphi_{2}^{n}\left(t\right)\right]\;+\delta^{i}\left(k_{12}^{n}R_{2}^{n}\left(R_{1}^{n}+R_{2}^{n}\right)\sin\varphi_{2}^{n}-k_{12}^{n}R_{1}^{n}R_{2}^{n}\sin\left(\varphi_{2}^{n}-\varphi_{1}^{n}\right)\right)+F_{2}^{n}\left(t\right).$$

here:

$\varphi_{j}^{i}\left(t\right)$—is the angular deflection of the *i*-th nitrogen base of the *j*-th chain counted counterclockwise at time *t*;

$I_{j}^{i}$—is the rotational inertia of the *i*-th nitrogen base of the *j*-th chain;

$R_{j}^{i}$—is the distance between the center of inertia of the *i*-th nitrogen base of the *j*-th chain to the sugar–phosphate chain;

$K_{j}^{i}$—is the constant characterizing the torsion moment of the *i*-th segment of the *j*-th sugar–phosphate chain;

$k_{12}^{i}$—is the constant characterizing the bond elastic properties of the *i*-th nitrogen base pairs;

$F_{j}^{i}\left(t\right)$—external influence on the *i*-th nitrogen base of the *j*-th chain at time *t*,

n—is the number of nitrogen base pairs in the system.

The magnitude of the external impact is $F_{j}^{i}\left(t\right)=-\beta_{j}^{i}\frac{d\varphi_{j}^{i}}{dt}\left(t\right)+F_{0}\cos\omega t$, where $-\beta_{j}^{i}\frac{d\varphi_{j}^{i}}{dt}\left(t\right)$ simulates the energy dissipation caused by the environment around the DNA molecule. $F_{0}\cos\omega t$ is an external periodic force.

In Equations (1)–(6), to the right of the equal sign, the first term is the influence of the sugar–phosphate chain on the *i*-th nitrogen base; the second term is the effect of the complementary nitrogen base; and the third term is the periodic external effect.

Thus, Equations (1)–(6) allow us to model the hydrogen bond in the *i*-th pair ($\delta^{i}=1$, $k_{12}^{i}=k_{12}^{H,i}$), deuterium ($\delta^{i}=1$, $k_{12}^{i}=k_{12}^{D,i}$), and the breaking of this bond ($\delta^{i}=0$). We will assume that a break in base pairs occurs if the potential binding energy in these pairs exceeds a certain critical value $E_{cr}^{H}$ for a hydrogen bond and $E_{cr}^{D}\;$ for a deuterium bond; if the potential energy in a pair with a broken bond is less than the critical value, then the bond is restored.

The initial conditions of Equations (1)–(6):

(7) $$\varphi_{1}^{i}\left(0\right)=\varphi_{1,0}^{i},\frac{d\varphi_{1}^{i}}{dt}\left(0\right)=\varphi_{1,1}^{i},$$

(8) $$\varphi_{2}^{i}\left(0\right)=\varphi_{2,0}^{i},\frac{d\varphi_{2}^{i}}{dt}\left(0\right)=\varphi_{2,1}^{i},i=\bar{1,n}.$$

We assume that at t=0 the system is in equilibrium, that is, at the initial conditions (7) and (8):

(9) $$\varphi_{1,0}^{i}=\varphi_{1,1}^{i}=\varphi_{2,1}^{i}=0,\varphi_{2,0}^{i}=\pi ,i=\bar{1,n}.$$

(1)–(8) is a Cauchy problem for a system of 2n ordinary differential equations. 

The influence of a single deuterium substitution for protium on the frequency generation of different-size bubbles was studied for the gene encoding interferon alpha 17. For this gene, n=980, the coefficient values for Equations (1)–(6) are taken from [53]), also $F_{0}=0.526\times 10^{-22}\;J,\;\omega =0.4\times 10^{12}\;s^{-1}$.

We assume that $E_{cr}^{D}=k^{D}\cdot E_{cr}^{H}$, $k_{12}^{D,i}=k^{D}\cdot k_{12}^{H,i}\;$ if one of the hydrogen bonds in the *i*-th base pair is replaced with deuterium, $k^{D}=1.05$ (the deuterium bond is 5% stronger than the hydrogen bond). The order of the critical energy $E_{cr}^{H}$ is consistent with the experimental data from the work.

Using the numerical solution of problems (1)–(3), we calculate the probability *P_l_* of the occurrence of denaturation of the bubbles with length *l* in the DNA molecule. To do this, on the time interval [0, *T*], we construct a set of points *t_j_* = *j*τ, j = $\bar{1,m}$, where τ = *T/m*. At *t* = *t_j_*, if the ratio $q_{j}^{l}$ of the number of base pairs with a broken bond contained in bubbles of length *l* to the total number of base pairs *n* is 0:1; then the probability *P_l_* is equal to the arithmetic mean over the points *t_j_* of these ratios (10):

(10) $$P_{l}=(\sum_{j=1}^{m}q_{j}^{l})/m.$$

Problems (1)–(8) were solved by the 4th-order Runge–Kutta method at *T* = 3.0 × 10^−10^ s, τ = 0.0001·10^−10^ s (was used the computer program, which has certificate of state registration No. 2021618296, Kuban State University, Krasnodar, Russia).

### 4.2. Approach for Studying the Influence of a Single Deuterium/Protium Substitution on the Dissociation of Hydrogen Bonds in Various Parts of IFNA17

The affiliation of each nucleotide to the I part (promoter: from 1st to 49th nitrogenous base pair), II part (coding region: from 50th to 619th nitrogenous base pair), or III part (termination sequence: from 620th to 980th nitrogenous base pair) of IFNA17 was determined due to its sequence (serial) number [90,91,92]. The different DNA bubbles were divided into four groups according to their sizes, which determine functions and lifetimes [6,60,61,68,71,93,94,95,96,97,98,99]: group 1 was from 2 to 4 nucleobases (small bubbles); group 2–from 5 to 10 nucleobases (metastable bubbles); group 3–from 11 to 30 nucleobases (large bubbles); and group 4–over 30 nucleobases (very large bubbles). Moreover, the open state (OS = 1 bps) occurrence frequencies were also contemplated in the IFNA17 throughout the energy range of 0.250·10^−22^ N·m (when every $P_{i}$ is more than 0.0 for all bubble groups and OS) to 0.600·10^−22^ N·m (when every $P_{i}$ equals 0.0 for all bubble groups and OS) with the change in energy always equal to 0.050·10^−22^ N·m. 

For each DNA bubble group and OS under varied energies, the nucleobases with $P_{i_{max}}$ and higher $P_{i}$ were selected for the “Maximum” range and their sum was $n_{max}$, and the nucleobases with $P_{i_{min}}$ and lower $P_{i}$ were selected as representatives for the “Minimum” range and their sum was $n_{min}$ [85]. According to the below-presented variant of the modified BJ algorithm adapted for DNA bubbles [86], every *P*_i_ of the bubbles or OS was arranged from $P_{i_{min}}$ to $P_{i_{max}}$ and their numbers were calculated in each gene part:

(1) *i* ϵ range “Maximum” (BJ-max):
  - if $P_{i_{max}}-\frac{1}{10}\left(P_{i_{max}}-P_{i_{min}}\right)\geq P_{0}+\frac{1}{2}\left(P_{i_{max}}-P_{0}\right)$ and $P_{i_{max}}>P_{0}\geq P_{i_{min}}\geq 0$:
    $$P_{i}\geq P_{i_{max}}-\frac{1}{10}\left(P_{i_{max}}-P_{i_{min}}\right)\Rightarrow n_{max}=\sum nP_{i};\;\mathrm{or}\;\mathrm{else}:$$
  - if $P_{i_{max}}-\frac{1}{10}\left(P_{i_{max}}-P_{i_{min}}\right)<P_{0}+\frac{1}{2}\left(P_{i_{max}}-P_{0}\right)$ and $P_{i_{max}}>P_{0}\geq P_{i_{min}}\geq 0$:
    $$P_{i}\geq P_{i_{max}}-\frac{1}{4}\left(P_{i_{max}}-P_{0}\right)\Rightarrow n_{max}=\sum nP_{i};$$
(2) *i* ϵ range “Minimum” (BJ-min):
  - if $P_{i_{min}}+\frac{1}{10}\left(P_{i_{max}}-P_{i_{min}}\right)\leq P_{0}-\frac{1}{2}\left(P_{0}-P_{i_{min}}\right)\;and$ $P_{i_{max}}\neq P_{i_{min}}$ > 0:
    $$P_{i}\leq P_{i_{min}}+\frac{1}{10}\left(P_{i_{max}}-P_{i_{min}}\right)\Rightarrow n_{min}=\sum nP_{i};\;\mathrm{or}\;\mathrm{else}:$$
  - if $P_{i_{min}}+\frac{1}{10}\left(P_{i_{max}}-P_{i_{min}}\right)>P_{0}-\frac{1}{2}\left(P_{0}-P_{i_{min}}\right)\;and$ $P_{i_{max}}\neq P_{i_{min}}$ > 0:
    $$P_{i}\leq P_{i_{min}}+\frac{1}{4}\left(P_{0}-P_{i_{min}}\right)\Rightarrow n_{min}=\sum nP_{i};\;\mathrm{or}\;\mathrm{else}:$$
  - if $P_{i_{min}}$ = 0 and $0<n_{g_{\mathrm{CSz}}}/n_{g}\;\leq \;1:$
    $$P_{i}=0\Rightarrow n_{\min}=n_{g_{\mathrm{CSz}}}\left(E_{cr}^{z}\right)\times {\left(1-n_{g_{\mathrm{CSz}}}\left(E_{cr}^{z}\right)/n_{g}\right)}^{2},$$
    where $n_{max}$ is the number of *i*, which were included in the range “Maximum”;$\;P_{i_{max}}$ is the highest $P_{i}$ for each bubble group or OS under specific $E_{cr}$ values; $n_{min}$ is the number of *i*, which were included in the range “Minimum”; $P_{i_{min}}$ is the lowest $P_{i}$ for each bubble group or OS under specific $E_{cr}$ values; $P_{0}$ is $P_{i}$ when all hydrogen bonds in DNA are ^1^H; $CS_{z}$ are the closed states of each bubble group or OS (*z* can be 1, 2, 3, or 4 bubble group, or OS): $P_{\mathrm{CSz}}=0.0$; $E_{cr}^{z}$ is critical energy, which has a value of 1 or more; $CS_{z}$: $0<n_{g_{\mathrm{CSz}}}/n_{g}\;\leq \;1$; $n_{x_{\mathrm{CSz}}}$ is the number of $P_{i}$ that equal 0.0 for each bubble group or OS in the *x* part of gene (*x* can be I, II or III part of IFNA17) under specific $E_{cr}^{z}$ values; $n_{g}$ is the number of nitrogenous base pairs in the whole gene; $n_{g_{\mathrm{CSz}}}$ is the total number of $P_{i}$ that equal 0.0 for each bubble group or OS in the whole gene under specific $E_{cr}^{z}$ values.

The contribution of the A–T/G–C ratio in forming different DNA bubbles and OS, when considering its influence on the occurrence frequencies of the H-bond dissociation, was studied according to the distribution of Adenine, Thymine, Guanine and Cytosine nucleotides in the promoter, coding region, and termination sequence of IFNA17 (Table 3).

### 4.3. Statistics

We used some statistical methods to extract the significance of differences among $n_{max}$ and $n_{min}$ of the three gene parts. Yates corrected chi-squared test ($\chi_{Yates}^{2}$) was applied for a 2 × 2 contingency table (where degrees of freedom $\left(\nu \right)=\left(2-1\right)\cdot \left(2-1\right)=1)$. As a short-cut, for a 2 × 2 table with the following entries (Table 4):

Where A and B are the rows according to the parts of the gene: I, II, or III; S is the column of $n_{min}$ of the range “Minimum”, or $n_{max}$ of the range “Maximum”; F is the column of the rest of the number of nucleotide pairs in the corresponding gene part (I, II, or III); *a* and *c* are $n_{min}$ of the range “Minimum”, or $n_{max}$ of the range “Maximum” in each compared part of the gene in the determined range of $E_{cr}$; *b* and *d* are equal to the total number of base pairs of the gene part minus $n_{min}$ of the range “Minimum”, or $n_{max}$ of the range “Maximum” in each compared part of the gene in the determined range of $E_{cr}$; $N_{A}=a+b;\;N_{B}=c+d;\;N_{S}=a+c;\;N_{F}=b+d;\;N=N_{A}+N_{B}+N_{S}+N_{F}$:

$$\chi_{Yates}^{2}=N{\left(\left|ad-bc\right|-N/2\right)}^{2}/N_{A}\cdot \;N_{B}\cdot N_{S}\cdot N_{F}.$$

Chi-square corrected by the procedure for the Bonferroni ($\chi_{B}^{2}$) was used for a 3 × 2 contingency table (where 3 is rows, 2 is columns, $\nu =\left(3–1\right)\cdot \left(2–1\right)=2)$. The Kruskal–Wallis ANOVA Ranks test (KWt) was applied for the comparison between different values for the determined range of $E_{cr}$ for the three gene parts that are mutually independent. To assess the relationship between the two variables, we utilized a Spearman’s rank correlation coefficient ($r_{\mathrm{Spearman}}$).

## 5. Conclusions

Thus, our mathematical modeling was developed for counting the different-size DNA bubble occurrences under varied energies without simplifications or averaging. This new approach demonstrated its utility under both conditions: when the $P_{i}$ of certain DNA bubbles is greater than 0.0 and when the $P_{i}$ of certain DNA bubbles is equal to 0.0. It is worth noting that, according to the obtained data, the possibility of the regulatory role of the non-coding region of IFNA17 in the DNA bubble formation even after a single ^2^H-substitution in the III part of the gene is shown. Different from the termination sequence, a single ^2^H/^1^H replacement in the promoter has the lowest influence on the molecular dynamics of all bubble groups in IFNA17. Moreover, our study clearly pointed out that, when a single ^2^H-substitution has taken place, the AT/GC ratio in forming DNA bubbles is dependent on several factors, such as the number of dissociated adjacent hydrogen bonds (positive correlation), $E_{cr}$ (inverse correlation for these bubble groups with the exception of the third), and $n_{\max}$ and $n_{\min}$ that create small bubbles with a higher proportion of AT than the bubbles of the same size formed by the nucleobases located out of the “Maximum” or “Minimum” ranges. In the A–T richest III part of the gene, there are higher percentages of ^2^H-substituted G–C bases, which abruptly decrease in occurrence in small (2–4 bps) and very large (over 30 bps) bubbles under energy values equal to 0.500 × $10^{-22}$ N·m and 0.300 × $10^{-22}$ N·m, respectively. And this can lead to a sharp slowdown in gene expression due to a significant rise in the non-dissociation of H-bonds in the promoter and coding regions. Also, it was revealed that positive values of bubble occurrence frequencies in IFNA17, in all cases and under the condition that all hydrogen bonds in base pairs are ^1^H, have an inverse relationship with DNA bubble size throughout the energy diapason from $0.250\times 10^{-22}$ N·m to $0.500\times 10^{-22}$ N·m. Such a result confirms the critical role of small and metastable bubbles in maintaining the functional capabilities of DNA (throughout the whole energy range) to initiate gene expression, during which the formation of bigger bubbles is required. This statement is reinforced by the outcome of the occurrence frequencies of small bubbles under the $0.250\times 10^{-22}$ N·m energy when they are even higher than the value of OS.

In addition, this study showed that a developed approach significantly decreases false positive results via a differentiated counting of the total sum of the $n_{\min}$ when parts of them have the $P_{i}$ equal to 0.0. Therefore, the variant of the modified BJ algorithm, which was adapted for DNA bubbles, can be helpful to study the regulatory mechanisms of DNA replication and transcription under different isotope substitutions in genes, and it can also be used to design certain DNA sequences that are more resistant to a single ^2^H/^1^H replacement (for example, when studying the effects of deuterium-depleted water or simulating the influence of Martian water, which is rich in deuterium compared to water on Earth [100,101,102]). Moreover, the BJ algorithm can be applied to studying the activity of DNA bubble generation in different genes and for various mathematical models (not only using a mechanical model of DNA). Further, such an approach can allow us to make recommendations when using deuterium/protium exchanges when creating treatments based on the modulating functional activity of genes, especially in patients with chronic severe pathology, such as cervical squamous cell carcinoma [34], chronic immune thrombocytopenia [33], prostate cancer [36], etc.

## Figures and Tables

**Table 1:** Total and A–T nucleobase quantities, forming the range “Maximum”, in different parts of IFNA17 after a single ^2^H/^1^H substitution.

$E_{cr}\cdot 10^{-22},\;N\cdot m$	Part of Gene	OS $(n_{max},\;n),$ [A–T bps]	$\mathrm{DNA}\;\mathrm{Bubble}\;\mathrm{Groups}\;(n_{max},\;n),\;[A–T\;\mathrm{bps}]$			
			1	2	3	4
0.250	I	0	0	0	0	1 [0]
	II	5 [1]	62 [26]	3 [2]	3 [0]	13 [6]
	III	3 [1]	9 [5]	0	0	2 [2]
0.300	I	0	0	0	0	0
	II	11 [1]	8 [1]	2 [0]	1 [1]	0
	III	2 [2]	0	0	0	34 [31]
0.350	I	0	0	0	0	-
	II	0	1 [0]	4 [2]	9 [3]	-
	III	2 [1]	0	34 [29]	0	-
0.400	I	0	0	0	0	-
	II	1 [0]	1 [0]	1 [0]	1 [0]	-
	III	0	0	0	0	-
0.450	I	0	0	0	-	-
	II	38 [13]	5 [0]	0	-	-
	III	1 [0]	0	3 [0]	-	-
0.500	I	0	0	-	-	-
	II	20 [5]	20 [5]	-	-	-
	III	0	0	-	-	-
0.550	I	27 [12]	-	-	-	-
	II	259 [121]	-	-	-	-
	III	10 [4]	-	-	-	-
0.600	I	-	-	-	-	-
	II	-	-	-	-	-
	III	-	-	-	-	-

Note: A is adenine, T is thymine, bps are base pairs, I is promoter, II is coding region, III is termination sequence, “-” is all nucleobases of the gene part that have $P_{i}$ equal to 0.0 for this option, OS is open state (1 bps), DNA bubble groups: 1 is 2–4 bps, 2 is 5–10 bps, 3 is 11–30 bps, and 4 is over 30 bps.

**Table 2:** Total and A–T nucleobase quantities, forming the range “Minimum”, in different parts of IFNA17 after a single ^2^H/^1^H substitution.

$E_{cr}\cdot 10^{-22},\;N\cdot m$	Part of Gene	OS $(n_{min},\;n),$ [A–T bps]	$\mathrm{DNA}\;\mathrm{Bubble}\;\mathrm{Groups}\;(n_{min},\;n),\;[A–T\;\mathrm{bps}]$			
			1	2	3	4
0.250	I	0	0	1 [1]	4 [1]	31 [20]
	II	30 [17]	21 [9]	39 [22]	9 [2]	300 [179]
	III	54 [39]	60 [41]	41 [31]	2 [2]	183 [130]
0.300	I	0	0	0	0	2 [1]
	II	8 [5]	9 [5]	2 [0]	5 [1]	18 [9]
	III	0	0	0	0	8 [5]
0.350	I	0	0	0	0	-
	II	24 [8]	10 [9]	79 [54]	2 [0]	-
	III	0	5 [2]	120 [97]	0	-
0.400	I	1 [0]	0	0	0	-
	II	33 [8]	3 [1]	33 [10]	62 [10]	-
	III	0	0	33 [9]	10 [3]	-
0.450	I	2 [0]	0	0	-	-
	II	34 [5]	0	7 [3]	-	-
	III	2 [0]	3 [0]	0	-	-
0.500	I	0	0	-	-	-
	II	100 [25]	62 [15]	-	-	-
	III	109 [69]	67 [43]	-	-	-
0.550	I	0	-	-	-	-
	II	3 [0]	-	-	-	-
	III	0	-	-	-	-
0.600	I	-	-	-	-	-
	II	-	-	-	-	-
	III	-	-	-	-	-

Note: A is adenine, T is thymine, bps are base pairs, I is promoter, II is coding region, III is termination sequence, “-” is all nucleobases of the gene part that have a $P_{i}$ equal 0.0 for this option, OS is open state (1 bps), DNA bubble groups: 1 is 2–4 bps, 2 is 5–10 bps, 3 is 11–30 bps, and 4 is over 30 bps.

**Table 3:** Distribution of adenine–thymine (A–T) and guanine–cytosine (G–C) base pairs in different parts of IFNA17.

Part of IFNA17	Nucleobase Pair Quantity	A–T (%)	G–C (%)
I (Promoter)	49 (from 1 to 49)	55.10	44.90
II (Coding region)	570 (from 50 to 619)	52.98	47.02
III (Termination sequence)	361 (from 620 to 980)	70.91	29.09

**Table 4:** Chi-squared test with Yates correction.

	S	F	
**A**	*a*	*b*	*N*_A_
**B**	*c*	*d*	*N*_B_
	*N*_S_	*N*_F_	*N*

## Data Availability

Not applicable.

## Supplementary Materials

The following supporting information can be downloaded at: https://www.mdpi.com/article/10.3390/ijms241512137/s1.
